# Long Noncoding RNA THAP9-AS1 and TSPOAP1-AS1 Provide Potential Diagnostic Signatures for Pediatric Septic Shock

**DOI:** 10.1155/2020/7170464

**Published:** 2020-12-01

**Authors:** Yong Wu, Qigai Yin, Xiaobao Zhang, Pin Zhu, Hengfei Luan, Ying Chen

**Affiliations:** ^1^Department of Anesthesiology, The First People's Hospital of Lianyungang, Lianyungang City, 222002 Jiangsu Province, China; ^2^Department of pediatrics, The First People's Hospital of Lianyungang, Lianyungang City, 222002 Jiangsu Province, China

## Abstract

**Background:**

Sepsis is a systemic inflammatory syndrome caused by infection with a high incidence and mortality. Although long noncoding RNAs have been identified to be closely involved in many inflammatory diseases, little is known about the role of lncRNAs in pediatric septic shock.

**Methods:**

We downloaded the mRNA profiles GSE13904 and GSE4607, of which GSE13904 includes 106 blood samples of pediatric patients with septic shock and 18 health control samples; GSE4607 includes 69 blood samples of pediatric patients with septic shock and 15 health control samples. The differentially expressed lncRNAs were identified through the limma R package; meanwhile, GO terms and KEGG pathway enrichment analysis was performed via the clusterProfiler R package. The protein-protein interaction (PPI) network was constructed based on the STRING database using the targets of differently expressed lncRNAs. The MCODE plug-in of Cytoscape was used to screen significant clustering modules composed of key genes. Finally, stepwise regression analysis was performed to screen the optimal lncRNAs and construct the logistic regression model, and the ROC curve was applied to evaluate the accuracy of the model.

**Results:**

A total of 13 lncRNAs which simultaneously exhibited significant differences in the septic shock group compared with the control group from two sets were identified. According to the 18 targets of differentially expressed lncRNAs, we identified some inflammatory and immune response-related pathways. In addition, several target mRNAs were predicted to be potentially involved in the occurrence of septic shock. The logistic regression model constructed based on two optimal lncRNAs THAP9-AS1 and TSPOAP1-AS1 could efficiently separate samples with septic shock from normal controls.

**Conclusion:**

In summary, a predictive model based on the lncRNAs THAP9-AS1 and TSPOAP1-AS1 provided novel lightings on diagnostic research of septic shock.

## 1. Introduction

Sepsis is a systemic inflammatory response syndrome caused by infection with an unacceptably high mortality and even long-term morbidity for many of those who survive [1]. According to the overseas epidemiological investigation, the case fatality rate of sepsis has exceeded that of myocardial infarction and become the main cause of death of noncardiac patients in intensive care units, and it is also the main cause of morbidity and mortality of children in the world [2–4]. Early recognition and diagnosis of sepsis is required to improve pediatric care and intervene before advanced organ dysfunction, and consequently prevent pediatric mortality and complications [5]. Considered the gold standard, blood culture is impractical in clinical, limited due to its time-consuming, laborious, and delay in optimal treatment period [6]. Although many biomarkers in sepsis including lactate, proinflammatory cytokines, chemokines, C-reactive protein, and procalcitonin have been identified [7], the diagnosis still lacks specificity because of the complicated, dynamic changes during severe sepsis and septic shock [8]. This study is aimed at identifying efficient and accurate diagnostic signatures for pediatric septic shock.

As the various cellular immune response to various types of infection have distinctive features, the role of gene expression analysis was gradually recognized in septic patients. Many researchers have identified molecular biomarkers of sepsis and suggested novel targets for new sepsis therapies [9, 10]. New diagnosis methods are still developed for sepsis, including microRNAs and long noncoding RNAs (lncRNAs) [11]. lncRNAs are a group of noncoding RNAs larger than 200 nt in length, which have been demonstrated that can participate in a large number of biological processes [12]. Recently, increasing evidences indicated that the abnormal expression of lncRNAs is closely associated with many inflammatory disorder diseases [13–17]. Many studies also demonstrated that lncRNAs play essential roles in sepsis. The downregulation of lncRNA CCL2 inhibits the inflammation response of macrophages in sepsis [18]. lncRNA GAS5 can promote podocyte injury in sepsis by inhibiting the expression of PTEN [19]. Silencing of lncRNA NEAT1 exerts potent suppressive effects on immunity in sepsis by promoting microRNA-125-dependent MCEMP1 downregulation [20]. However, studies about the potential application of lncRNA in the diagnosis of pediatric septic shock are very lack.

In this study, we used the bioinformatics method to screen the potential lncRNAs which might possibly lead to pediatric septic shock and construct the classification model to provide early diagnosis for pediatric septic shock. Further, 13 lncRNAs were identified as potentially related to the occurrence of septic shock. The logistic regression model constructed in this study could efficiently separate the samples with or without septic shock and exerted a certain practical value for the diagnosis of pediatric septic shock.

## 2. Materials and Methods

### 2.1. Data Collection

The two mRNA profiles GSE13904 [21] and GSE4607 [22] were downloaded from the Gene Expression Omnibus (GEO,https://www.ncbi.nlm.nih.gov/geo/), of which GSE13904 includes 106 blood samples of children with septic shock and 18 control samples of healthy children; GSE4607 includes 69 blood samples of children with septic shock and 15 control samples of healthy children. The two expression profiles were all detected by the Affymetrix Human Genome U133 Plus 2.0 Array.

### 2.2. Differential Expression Analysis

The expression profiles of lncRNA from the two data above were extracted, and the probes with missing values were removed; then, standardization was performed based on the robust multiarray (RMA) method. Subsequently, the differential expression analysis of lncRNA was performed by using the limma function package of the R language [23], with ∣log 2 (fold change (FC)) | >1 and *p* ≤ 0.05 as the significant threshold.

### 2.3. Functional Enrichment Analysis

Gene Ontology (GO) analysis (including biological process, molecular function, and cellular component) and Kyoto Encyclopedia of Genes and Genomes (KEGG) pathway enrichment analysis were performed by using the clusterProfiler function package of the R language [24], and *p* < 0.05 was considered as the threshold.

### 2.4. Protein–Protein Interaction Networks

The STRING database (https://string-db.org/, version 11.0) is a database which is used to analyze and predict the functional connections and interactions of proteins [25]. Here, the STRING database was applied and the interaction pairs of proteins with confidence score ≥ 0.4 are retained. The PPI network was visualized based on Cytoscape (https://cytoscape.org/, version 3.7.2) [26]. Meanwhile, the key clustering modules were screened based on the molecular complex detection method (MCODE) plug-in of the Cytoscape software, with MCODE score > 4 as the significant threshold.

### 2.5. The Construction of the Logistic Regression Model

The glmnet function in R language [27] was used to construct the multivariate logistic regression model with the expression value of lncRNA as the continuous predictive variable and the sample type as the categorical responsive value (septic shock or not), and the receiver operating characteristics (ROC) analysis was performed to evaluate the accuracy of the model.

## 3. Results

### 3.1. Identification of Differentially Expressed lncRNAs

We first extracted the lncRNA profiles from two databases and standardized the expression profiles. The results showed that there was no obvious change in the deviation of each sample from two datasets (Figure S1A and B), suggesting it could be used for subsequent analysis. To further confirm the repeatability of the data within the group, principal component analysis (PCA) was analyzed based on the expression value of lncRNAs, and the results indicated that the case group (pediatric septic shock) and control group (healthy children) could be efficiently separated (Figure S1C and D), suggesting a better reproducibility of data in the group.

**Figure 1:**
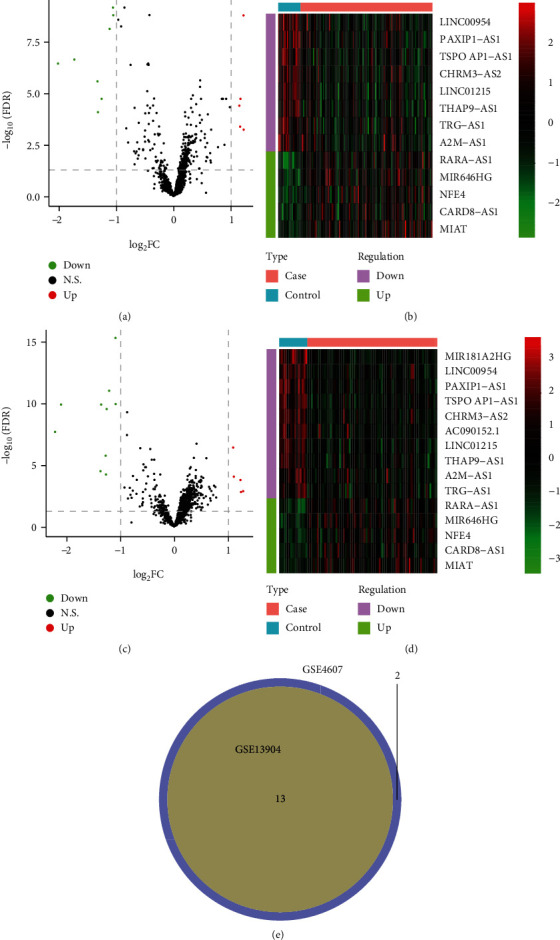
The identification of differentially expressed lncRNAs. (a) The volcano plot of differentially expressed lncRNAs between the case group and the control group in GSE13904. The horizontal axis is Log2 FC, and the vertical axis is -log10 (FDR). The red points represent the upregulated lncRNAs, the blue points represent the downregulated lncRNAs, and the black points indicate no significant difference. (b) The heat map of differentially expressed lncRNAs between the case group and the control group in GSE13904.

Then, differential expression analysis was performed; for GSE13904, 13 differentially expressed lncRNAs (5 upregulated and 8 downregulated) were identified in the case group compared with the control group (Figure 1(a)), and the expression of 13 lncRNAs all had significant different expressions between the two groups (Figure 1(b)). For GSE4607, a total of 15 differentially expressed lncRNAs (5 upregulated and 10 downregulated) were identified in the case group compared with the control group (Figure 1(c)), and the expression of the 15 lncRNAs all had significantly different expressions between the two groups (Figure 1(d)). In addition, there were 13 lncRNAs (LINC00954, PAXIP1-AS1, RARA-AS1, TSPOAP1-AS1, CHRM3-AS2, LINC01215, THAP9-AS1, TRG-AS1, MIR646HG, NFE4, A2M-AS1, CARD8-AS1, and MIAT) which simultaneously exhibited significant differences in the case group compared with the control group from two sets (Figure 1(e)), indicating that these 13 lncRNAs might be key lncRNAs that led to septic shock in children.

### 3.2. Functional and Pathway Enrichment Analysis

**Figure 2:**
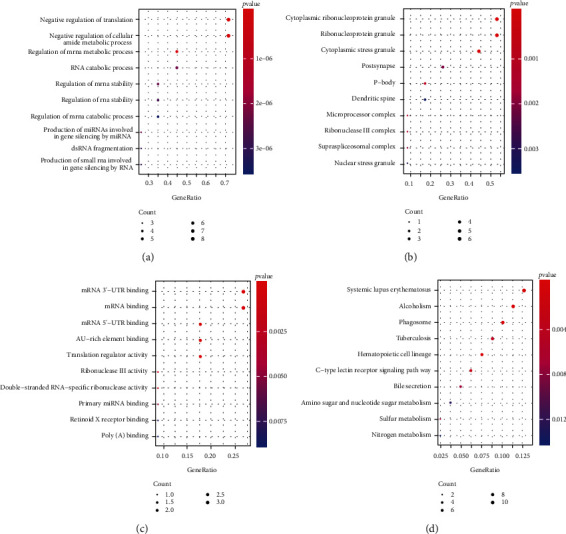
The enrichment of GO terms and KEGG pathways based on 13 lncRNAs. (a)-(c) The top 10 most significantly enriched BP (a), CC (b), and MF (c) terms. (d) The top 10 most significantly enriched KEGG pathways. The horizontal axis represents the GeneRatio (enrichment ratio), and the vertical axis indicates the corresponding biological process or KEGG pathway. The larger the dot is, the more genes are enriched, and the color of the dot corresponds to the *p* value.

To explore the metabolic pathways closely involved in the occurrence of septic shock in children, the target genes of 13 lncRNAs were predicted by using starBase (http://starbase.sysu.edu.cn/,version 2.0) [28]. The results showed that a total of 18 target genes including FUS, IGF2BP1, PUM2, EIF4A3, DGCR8, LIN28B, LIN28A, CAPRIN1, FUS-mutant, TAF15, U2AF2, TIA1, TIAL1, HNRNPC, UPF1, IGF2BP3, PTBT1, and TARDBP were predicted. Then, functional and pathway enrichment analysis was performed, and the results suggested that there were 98 significantly enriched biological process (BP) terms including the regulation of mRNA or RNA stability, RNA silencing, mRNA catabolic process and cytokine biosynthetic process (*p* < 0.05), 17 significantly enriched cellular component (CC) terms including cytoplasmic stress/ribonucleoprotein/ribonucleoprotein granule (*p* < 0.05), and 30 significantly enriched molecular function (MF) terms including mRNA 3'-UTR/5'-UTR binding, translation regulator activity, and catalytic activity on RNA (*p* < 0.05), as well as 24 significantly enriched KEGG pathways including Epstein-Barr virus infection, primary immunodeficiency, and T cell receptor signaling pathway (*p* < 0.05). The full list of significantly enriched GO terms and KEGG pathways is shown in Table S1. Meanwhile, the top 10 most significantly enriched BP, CC, and MF terms are shown in Figures 2(a)-2(c), and the top 10 most significantly enriched KEGG pathways are shown in Figure 2(d). Besides, enrichment analysis of these 18 target genes was also performed by STRING based on Reactome Pathways, UniProt, and InterPro databases. The significantly enriched Reactome Pathways has 9 entries, as shown in Table 1. As shown in Table S1, there were 20 significantly enriched UniPort items and 16 significantly enriched InterPro items.

### 3.3. The Construction of PPI Network

**Figure 3:**
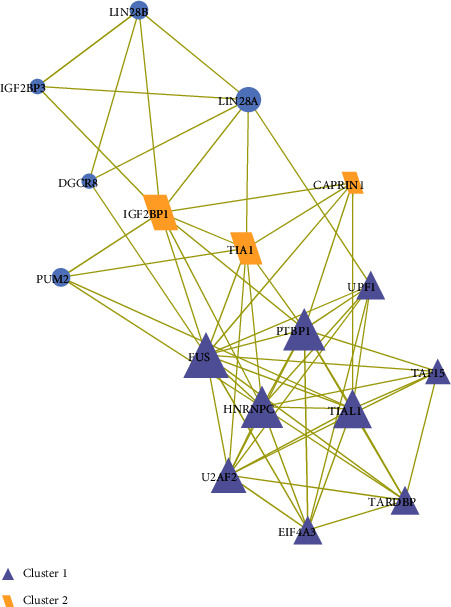
The construction of the PPI network. Each dot represents a node. The more lines connected with the dot, the greater the degree of the node, and the more important the lncRNA in the network. The size of the nodes is used to visually reflect the degree size, and the thicker the line, the stronger the interaction between the two nodes. The unique shapes and colors represent different modules, with the purple triangle for cluster 1 and the orange parallelogram for cluster 2.

Next, the PPI network was constructed based on the 18 target genes; then, the interaction pairs of proteins of which confidence score ≥ 0.4 were selected and visualized using the Cytoscape software (Figure 3). We found that there were 17 interactional genes with the maximum node degree of FUS at 12, and the minimum node degree is 1. Meanwhile, the two significant clustering modules with MCODE score > 4 were identified based on the MCODE plug-in: cluster 1 included FUS, PTBT1, UPF1, HNRNPC, U2AF2, TIAL1, EIF4A3, and TARDBP, and cluster 2 includes IGF2BP1, TIA1, and CAPRIN1. The results suggested that these 11 genes might be key factors which were close to the occurrence of septic shock in children.

### 3.4. The Construction of Logistic Regression Model

Finally, the logistic regression model was constructed based on the 13 lncRNAs which were all had significant difference in the case group compared with control group in the two data sets. We randomly selected 82 samples as the training set from GSE13904 to construct logistic regression model with expression value of 13 lncRNAs as the continuous type prediction variable and the sample type (septic shock or not) as the categorical response variable. Meanwhile, the remaining samples of GSE13904 were used as the testing set, and the samples of GSE4607 were used as an independent validation set to verify the effect of the model.

**Figure 4:**
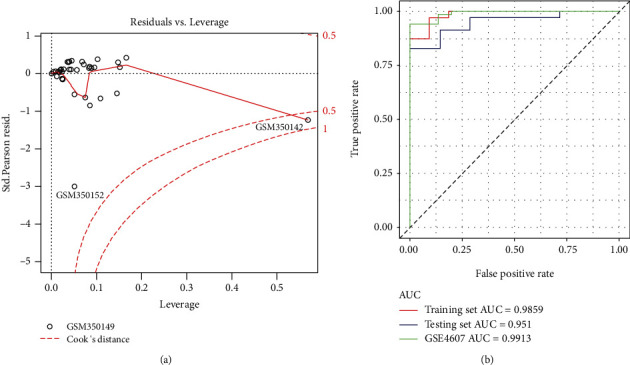
The construction of logistic regression diagnostic model. (a) The logistic regression diagnostic model. The red dashed line represents the COOK distance. Generally, points with COOK distance > 0.5 may affect the accuracy of the model. (b) The ROC curve of the logistic regression diagnostic model. The AUC value is the area under the curve and can intuitively evaluate the quality of the model; the larger the AUC value, the better the model.

In order to construct the model with strong interpretation with as few lncRNAs as possible, stepwise regression analysis was performed and screened two optimal lncRNAs THAP9-AS1 and TSPOAP1-AS1. Then, the final logistic regression model was constructed based on THAP9-AS1 and TSPOAP1-AS1 (Figure 4(a)), and the detailed parameters of the model are shown in Table 2, of whichodds ratio (OR) > 1suggested that the expression of lncRNA was positively correlated with the occurrence of septic shock andOR < 1indicating a negative correlation. The results indicated that the expression of THAP9-AS1 and TSPOAP1-AS1 were all negatively correlated with the occurrence of septic shock, suggesting that the low level of THAP9-AS1 and TSPOAP1-AS1 was more likely to lead to septic shock. Meanwhile, there was a sample that might have little impact on the accuracy of the model (GSM350142, COOK distance > 0.5). The accuracy of the model was evaluated by the ROC curve (Figure 4(b)) and showed that the area under curve (AUC) value in the training set and testing set of GSE13904 was 0.9859 and 0.951, respectively. Moreover, the AUC value of the validation set in GSE4607 was 0.9913. These results showed that the logistic regression model constructed based on THAP9-AS1 and TSPOAP1-AS1 could efficiently distinguish samples with or without septic shock, suggesting it might be potentially applied to the diagnosis for pediatric septic shock.

## 4. Discussion

Septic shock is one of the main causes of mortality even in children [29]. The treatment of severe sepsis and septic shock is described by the Surviving Sepsis Campaign including early recognition, microbial source control, rapid and appropriate treatment with antimicrobial agents, and goal-directed haemodynamic, ventilator, and metabolic therapies [30]. When sepsis is not treated correctly and quickly, all organs can be affected, and each developing organ failure increases the risk of mortality [31]. Therefore, the identification of efficient diagnostic makers for the prevention and treatment of septic shock is still urgent. It has been reported that lncRNAs have been identified as predictive biomarkers for the diagnosis, severity, and prognosis of patients with sepsis [32, 33]. However, the diagnostic value of lncRNAs in pediatric septic shock has been not reported. In the present study, we identified 13 potentially risk lncRNAs (LINC00954, PAXIP1-AS1, RARA-AS1, TSPOAP1-AS1, CHRM3-AS2, LINC01215, THAP9-AS1, TRG-AS1, MIR646HG, NFE4, A2M-AS1, CARD8-AS1, and MIAT) which might lead to septic shock.

Annane et al. have reviewed that pathogens trigger sequential intracellular events in immune cells, epithelium, endothelium, and the neuroendocrine system through their microbial-associated molecular patterns and proinflammatory mediators which contribute to the eradication of invading microorganisms are produced, and anti-inflammatory mediators control this response [34]. The inflammatory response leads to damage to host tissue, and the anti-inflammatory response causes leucocyte reprogramming and changes in immune status [35]. Hence, to determine which functions or pathways were involved in the occurrence of septic shock, the potential targets of lncRNAs were predicted based on the starBase. The GO and pathway enrichment analysis was performed by using the 18 targets and indicated that multiple immune-related functions or pathways such as cytokine biosynthetic/metabolic process, primary immunodeficiency, antigen processing/presentation, and Epstein-Barr virus infection were significantly enriched. These results confirmed that the immune damage induced by infection was the major cause of septic shock.

Within cells, proteins function through protein-protein interactions (PPI), which is essential for almost all biochemical activities to achieve specific tasks in life [36]. PPI also endows a single protein with multiple functions [37], and investigations on PPI methodologies and applications to disclosing mechanisms of biological processes draw increasing attention [38, 39]. Therefore, the PPI network was constructed based on the 18 targets, and the key clustering modules were screened based on the MCODE plug-in of the Cytoscape software. The results further suggested that the 11 genes including FUS, PTBT1, UPF1, HNRNPC, U2AF2, TIAL1, EIF4A3, TARDBP, IGF2BP1, TIA1, and CAPRIN1 might be key risk factors involved in the occurrence of septic shock.

In order to construct the logistic regression model with strong interpretation with as few lncRNAs as possible, stepwise regression analysis was performed and screened two optimal lncRNAs THAP9-AS1 and TSPOAP1-AS1. Meanwhile, the expressions of THAP9-AS1 and TSPOAP1-AS1 were all negatively correlated with the occurrence of septic shock, suggesting that low levels of THAP9-AS1 and TSPOAP1-AS1 were more likely to lead to septic shock. Although the effect of the two lncRNAs in sepsis remains unclear, their roles have been well studied in various human diseases. THAP9-AS1, induced by *Helicobacter pylori*, can promote cell growth and migration of gastric cancer [40]. THAP9-AS1 can also promote pancreatic ductal adenocarcinoma growth and lead to a poor clinical outcome via sponging miR-484 and interacting with YAP [41]. TSPOAP1-AS1 negatively modulated the (influenza A virus) IAV-induced Ifnb1 transcription, interferon-sensitive response element (ISRE) activation, and downstream interferon-stimulated gene expression, which suggested that TSPOAP1-AS1 could be efficiently utilized by viruses to support its replication [42]. In addition, TSPOAP1-AS1 was identified as biomarkers for pancreatic cancer based on the weighted gene coexpression network analysis (WGCNA) [43]. Finally, the logistic regression model was constructed using the optimal lncRNAs THAP9-AS1 and TSPOAP1-AS1 and could efficiently separate samples with or without septic shock. Moreover, the AUC value of the ROC curve further determined that the logistic regression model might potentially be applied to the diagnosis of pediatric septic shock.

## 5. Conclusion

In a word, our study identified 13 lncRNAs which were potentially involved in the occurrence of septic shock in children. The logistic regression model was constructed based on the optimal lncRNAs THAP9-AS1 and TSPOAP1-AS1 and could efficiently distinguish the samples with or without septic shock, which provided potentially diagnostic signatures for septic shock. In addition, THAP9-AS1 and TSPOAP1-AS1 had been proved to be involved in the immune response to regular various diseases; thus, they are likely to regulate disease progression by participating in the immune reaction of septic shock. In future research, THAP9-AS1 and TSPOAP1-AS1 could be verified in the diagnosis and intervention of pediatric septic shock.

## Figures and Tables

**Table 1:** The enriched terms of reactome pathways.

Term	Description	Count	Genes	*p* value
HSA-8953854	Metabolism of RNA	8	FUS, IGF2BP3, EIF4A3, IGF2BP1, U2AF2, PTBP1, HNRNPC, UPF1	1.78e-06
HSA-72163	mRNA splicing—major pathway	5	FUS, EIF4A3, U2AF2, PTBP1, HNR, NPC	1.14e-05
HSA-6803529	FGFR2 alternative splicing	3	PTBP1, TIAL1, TIA1	2.35e-05
HSA-428359	Insulin-like growth factor-2 mRNA binding proteins (IGF2BPs/IMPs/VICKZs) bind RNA	2	IGF2BP3, IGF2BP1	7.08e-05
HSA-72187	mRNA 3'-end processing	2	EIF4A3, U2AF2	0.0076
HSA-109688	Cleavage of growing transcript in the termination region	2	EIF4A3, U2AF2	0.0091
HSA-159236	Transport of mature mRNA derived from an intron-containing transcript	2	EIF4A3, U2AF2	0.0091
HSA-73856	RNA polymerase II transcription termination	2	EIF4A3, U2AF2	0.0091
HSA-975957	Nonsense-mediated decay (NMD) enhanced by the exon junction complex (EJC)	2	EIF4A3, UPF1	0.0185

**Table 2:** Model interpretation of logistic regression model.

Gene	*β*	SE	OR	95% OR	*p*
THAP9-AS1	-1.045	0.6907	0.3517	0.0686-1.1381	0.1303
TSPOAP1-AS1	-2.7969	1.2138	0.061	0.0029-0.4402	0.0212

## Data Availability

The datasets (GSE13904 and GSE4607) for this study can be found in the (GEO) (https://www.ncbi.nlm.nih.gov/geo/).

## Supplementary Materials

Supplementary Materials Fig. S1 The standardization of lncRNA profiles and PCA analysis. (A) The boxplot of expression of lncRNA after standardization in GSE13904 data set. (B) The boxplot of expression of lncRNA after standardization in the GSE4607 data set. The horizontal axis represents the sample, and the vertical axis represents the relative expression of lncRNA. (C) The PCA analysis of lncRNA in the GSE13904 data set. (D) The PCA analysis of lncRNA in the GSE4607 data set. The points with different colors represent samples from different groups, and the closer the distance between the two points, the more similar the expression of lncRNA in the samples.
