# Supplementary material for: Long Noncoding RNA THAP9-AS1 and TSPOAP1-AS1 Provide Potential Diagnostic Signatures for Pediatric Septic Shock
Source: Biomed Res Int. 2020 Dec 1;2020:7170464. doi: 10.1155/2020/7170464 (PMC7725549; doi:10.1155/2020/7170464)
Supplement: Supplementary Materials — Fig. S1 The standardization of lncRNA profiles and PCA analysis. (A) The boxplot of expression of lncRNA after standardization in GSE13904 data set. (B) The boxplot of expression of lncRNA after standardization in the GSE4607 data set. The horizontal axis represents the sample, and the vertical axis represents the relative expression of lncRNA. (C) The PCA analysis of lncRNA in the GSE13904 data set. (D) The PCA analysis of lncRNA in the GSE4607 data set. The points with different colors represent samples from different groups, and the closer the distance between the two points, the more similar the expression of lncRNA in the samples. [file 7170464.f1.docx]

Supplementary Material

Fig. S1 The standardization of lncRNA profiles and PCA analysis. (A) The boxplot of expression of lncRNA after standardization in GSE13904 data set. (B) The boxplot of expression of lncRNA after standardization in GSE4607 data set. The horizontal axis represents the sample and the vertical axis represent the relative expression of lncRNA. (C) The PCA analysis of lncRNA in GSE13904 data set. (D) The PCA analysis of lncRNA in GSE4607 data set. The points with different colors represent samples from different groups, and the closer the distance between two points, the more similar the expression of lncRNA in the samples.

Table S1 The full list of significantly enriched GO terms and KEGG pathways.
